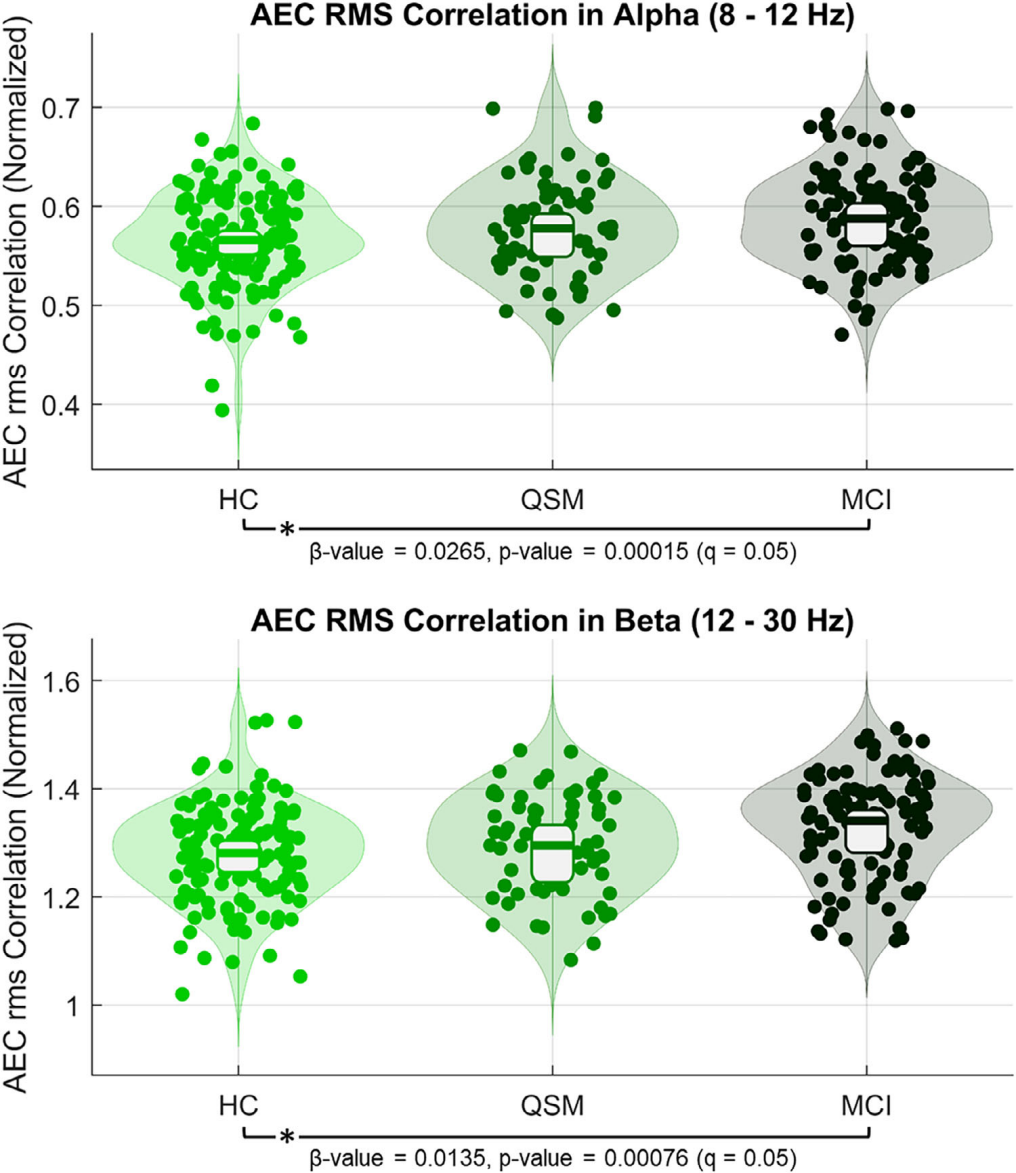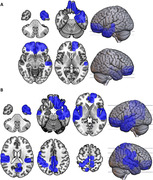# Dynamic functional connectivity evolution across the Alzheimer's Disease spectra

**DOI:** 10.1002/alz.092220

**Published:** 2025-01-09

**Authors:** Martín Carrasco‐Gómez, Jesús Cabrera, Alejandra García‐Colomo, Ricardo Bruña Fernández, Fernando Maestú, Andrés Santos, Center for Cognitive and Computational Neuroscience

**Affiliations:** ^1^ Biomedical Research Networking Center in Bioengineering Biomaterials and Nanomedicine (CIBER‐BBN), Madrid, Madrid Spain; ^2^ Universidad Politécnica de Madrid, Madrid, Madrid Spain; ^3^ Center for Cognitive and Computational Neuroscience, Madrid, Madrid Spain; ^4^ Center for Cognitive and Computacional Neuroscience, Madrid, Madrid Spain; ^5^ Universidad Complutense de Madrid, Madrid Spain; ^6^ Universidad Complutense de Madrid, Madrid, Madrid Spain; ^7^ Department of Experimental Psychology, Cognitive Processes and Speech Therapy, Universidad Complutense de Madrid, Madrid Spain

## Abstract

**Background:**

Recent studies in brain functional connectivity (FC) have shifted focus to dynamic functional connectivity (dFC), exploring transient aspects of FC over time. This shift is particularly relevant for Alzheimer's Disease (AD), as it involves altered cognition‐supporting networks. Our study aims to characterize the evolution of dFC across the entire pre‐dementia AD spectrum using Amplitude Envelope Correlation (AEC) recurrence matrices and to link this to cognitive decline.

**Method:**

We analyzed 143 healthy controls (HC), 72 subjective cognitive decline (SDC) participants, and 106 Mild Cognitive Impairment (MCI) patients. Leakage‐corrected AEC was computed in alpha (8‐12 Hz) and beta (12‐30 Hz) bands from resting eyes‐closed magnetoencephalography (MEG) signals. Recurrence matrices representing dFC stability over time were obtained by correlating AEC matrices over signal segments. Between‐group differences were tested via linear regression, controlling for sex and age. Group differences were further explored with cluster‐based permutation tests (CBPT), and MCI whole‐brain stability was correlated with mini mental state evaluation (MMSE) scores. All results are FDR corrected (q = 0.05).

**Results:**

Significant decreases in dFC stability between the HC and MCI groups were observed in both alpha and beta bands (Figure 1, alpha: p‐value < 0.001, β‐value = 0.0265; beta: p‐value < 0.001, β‐value = 0.0135). CBPT analyses identified significant differences in orbitofrontal and left temporal cortices in alpha (Figure 2A; p = 0.028), and in the left orbital and temporal regions and in several areas related to the Default Mode Network (DMN), including the hippocampus and precuneus in beta (Figure 2B; p = 0.009). Additionally, a significant inverse correlation between MCI whole‐brain stability and MMSE scores was found in both frequency bands (alpha: R = ‐0.27, p‐value = 0.0084; beta: R = ‐0.26, p‐value = 0.0117).

**Conclusion:**

Our findings suggest a decrease in dFC dynamics along the AD continuum, particularly affecting orbitofrontal and temporal regions, as well as DMN‐related areas like the hippocampus and inferior parietal gyrus. These changes are inversely correlated with cognitive performance in MCI patients. This indicates that dFC parameters derived from AEC recurrence matrices could be valuable in identifying early AD progression.